# The Effects of Sex and Chronic Restraint on Instrumental Learning in Rats

**DOI:** 10.1155/2013/893126

**Published:** 2013-02-28

**Authors:** Angela L. McDowell, Kathryn M. Heath, Preston E. Garraghty

**Affiliations:** ^1^Department of Psychological and Brain Sciences, Indiana University, Bloomington, IN 47405, USA; ^2^Program in Neuroscience, Indiana University, Bloomington, IN 47405, USA; ^3^Postdoctoral Associate, Department of Medicine, University of Pittsburgh, 628 NW MUH, 3459 5th Avenue, Pittsburgh, PA 15213, USA

## Abstract

Chronic stress has been shown to impact learning, but studies have been sparse or nonexistent examining sex or task differences. We examined the effects of sex and chronic stress on instrumental learning in adult rats. Rats were tested in an aversive paradigm with or without prior appetitive experience, and daily body weight data was collected as an index of stress. Relative to control animals, reduced body weight was maintained across the stress period for males (−7%, *P* ≤ .05) and females (−5%, *P* ≤ .05). For males, there were within-subject day-by-day differences after asymptotic transition, and all restrained males were delayed in reaching asymptotic performance. In contrast, stressed females were facilitated in appetitive and aversive-only instrumental learning but impaired during acquisition of the aversive transfer task. Males were faster than females in reaching the appetitive shaping criterion, but females were more efficient in reaching the appetitive tone-signaled criterion. Finally, an effect of task showed that while females reached aversive shaping criterion at a faster rate when they had prior appetitive learning, they were impaired in tone-signaled avoidance learning only when they had prior appetitive learning. These tasks reveal important nuances on the effect of stress and sex differences on goal-directed behavior.

## 1. Introduction

The effects of chronic stress on learning and memory in rats have most commonly been investigated using three weeks of daily restraint (e.g., [1]). In general, experiments using the three weeks of restraint model have most commonly examined males and employed spatial learning or fear conditioning tasks [2–5]. Three weeks of restraint enhanced freezing in males while simultaneously impairing recall [2] and acquisition memory [3] in the Morris water maze (MWM) [2]. In contrast, multiple studies report minimal impact [4] to no change [6] or facilitation of learning in females [2, 6]. Given that human females are twice as likely to suffer from disorders of chronic stress [7, 8] and that reports of task facilitation dominate the, albeit much smaller, literature for female rats, this begs the issue of task selection and how to integrate these seemingly disparate datasets from human females to animal models.

Previous studies using instrumental learning tasks have shown an interaction effect of sex by motivation type [9], which is one potential substrate to examine possible sex by task differences. Studies generally indicate that chronic stress enhances instrumental responding to pleasant stimuli [10] in both male and female rats but is duration dependent [11], while it differentially impacts instrumental responding to aversive stimuli depending upon stressor onset in male rats. For example, studies have found sex by task motivation demand differences in instrumental learning; while the males met appetitive criterions faster, females were more efficient in aversive tasks [9]. However, relatively little attention has been directed at investigating the effects of chronic stress on instrumental learning. An older study reported that stress prior to the onset of conditioning had no effect on acquisition [11], whereas when stress and conditioning onset were concurrent, or when stress followed acquisition, learning or recall was impaired. Unfortunately, this study involved only two weeks of uncontrollable and unpredictable footshock stress in male subjects, so it is difficult to compare it to the results from studies using the 3-week restraint model. A more recent study examined the effect of three weeks of restraint following conditioning on appetitive learning and found decreased appetitive motivation in males [12]. There is no available data on instrumental responding to aversive stimuli in chronically restrained females and no investigations of possible sex differences in instrumental learning as a consequence of chronic restraint stress. 

In the present experiments, we have used two instrumental learning paradigms to test for sex differences in chronically restrained male and female rats. Pavlovian instrumental learning requires the subject to make an association between two stimuli and subsequently volitionally manipulates an object as an indication of choice, typically a lever or barpress. The animals were restrained for 3 weeks and then tested in either an active avoidance task or in an appetitive-to-aversive transfer task, paradigms that we have previously employed in both male and female rats (e.g., [13–16]). This paradigm was selected because of its ability to test lower level associations (i.e., shaping and nontransfer-signaled learning) as well as the ability to test discrimination learning (i.e., appetitive-to-aversive transfer learning). The goals of this study are to (1) compare the effects of chronic stress on rats on Pavlovian instrumental learning and (2) to compare the effects of chronic stress between male and female rats on learning. 

## 2. Methods

### 2.1. Subjects

Adult Sprague-Dawley rats weighing between 175–275 g (females) and 250–350 g (males) were maintained at 85% of their free-feeding weight throughout all phases of instrumental training in order to motivate animals during appetitive learning. Due to food restriction, all animals were housed individually. Weight assessments were made immediately prior to training, and subsequently, the animal was returned to its home tub with access to food. All animals began food restriction 5 days before the end of the restraint period. Day 1 of appetitive shaping began immediately following the last day of restraint. The rats had access to water *ad libitum* in their home cage and were housed in a 12-hour on: 12-hour off lighting environment with testing having occurred during the light phase. All procedures were done ethically and approved by the Indiana University Institutional Animal Care and Use Committee (IACUC).

### 2.2. Stress Apparatus

#### 2.2.1. Chronic Restraint

Animals were transported to a separate room and restrained for six hours per day between the hours of 10 am and 4 pm, seven days/week, and for 21 consecutive days. The restraining devices were made in house and were adjustable to fit the rat, so that the movement was eliminated, but injury did not occur. The restrainers were made of Plexiglas and encircled the rat, with adjustable slots for length, circumference, and width. There was a stainless steel, nondetachable mesh wire at the nose end of the restrainer, which allowed for adequate air flow and an adjustable stainless steel piece at the tail end. The rats were guided into the tail end of the restrainer, and the stainless steel piece was lowered behind the rat to secure its restraint. All restrained rats were removed from their home cages and taken to a separate room during the restraining time period and were returned following daily restraint. Handling of the control animals cooccurred with the onset of food restriction five days prior to shaping and consisted of five minutes of gentle petting. All animals were housed within the same colony with same sex littermates and were individually housed at 60 days of age and therefore sexually naïve.

Some of the animals were tested in the appetitive-to-aversive transfer task (AP-AV): restrained females (*n* = 14), control females (*n* = 16), restrained males (*n* = 6), and control males (*n* = 9). The remaining animals were tested in the aversive task without prior appetitive training (AV): restrained females (*n* = 11), control females (*n* = 17), restrained males (*n* = 8), and control males (*n* = 9). 

### 2.3. Body Weight Assessment

Additional male and female litter-matched animals were assigned to either 21 days of chronic restraint from 10 am to 4 pm (female, *n* = 5; male, *n* = 5) or to control group (female, *n* = 5; male, *n* = 5). Animals in both groups (*n* = 20) were weighed daily as the whole body weight has been shown to provide a valid index of stress (e.g., Bassett and Cairncross [17]).  Figure 6 lays out the timeline of the progression of all rats through the protocol.

### 2.4. Learning Procedures

#### 2.4.1. Appetitive Paradigm (AP)

For appetitive learning, subjects were shaped using a method of successive approximations to barpress for food reward (sugar pellet), which began on the day immediately following the last day of restraint. When the rat pressed the bar 100 times within 30 min on a fixed ratio (FR-1) schedule (for one day only), it was then advanced to a fixed ratio 4 (FR-4) schedule of reinforcement. Animals were required to produce 100 reinforced lever presses within 40 min over two consecutive days in order to advance to tone training in which a 3-second tone (2000 Hz, 90 dB SPL) signaled the availability of reinforcement. Reinforcement was contingent upon a lever press during the 3-second tone period. A single tone-signaled training session consisted of 100 trials. Each trial began with a randomly determined 1–8 sec pretone period followed by the tone onset which lasted 3 sec or until the lever was pressed. Tone offset initiated a 15-sec intertrial interval (ITI). If the rats pressed the bar during the pretone period, the period was reset, and the trial was delayed until no barpresses occurred during the randomly determined pretone period. Appetitive training continued until the rat achieved a 90% correct response (CR) rate or higher (i.e., barpressing during the tone) for two consecutive days. 

#### 2.4.2. Aversive Paradigm (AV)

On the day immediately following appetitive training, transfer animals began the aversive learning task, which occurred within the same experimental chamber that appetitive training had occurred. Additional rats were placed directly into the aversive learning task with no prior appetitive experience. In order to directly compare the effect of restraint between transfer and AV-only animals, the average amount of time transfer animals spent in the appetitive paradigm was estimated and AV-only animals waited this amount of time after restraint cessation before beginning aversive shaping (average = 7 days). The aversive context consisted of a shock that could be escaped or avoided with a lever press. The shock intensity was maintained at 0.8–1.0 mA. The animals were introduced to the aversive context in a single training session where shock pulses (250 ms at 1.33 Hz) were presented continuously until the bar was pressed. If the animal did not press the bar within 30 pulses, the current was turned off manually for a rest period of 10 sec. When the animal pressed the bar, a rest period of 30 sec was initiated. Shaping criterion was 15 consecutive escape responses after no more than 5 shock pulses. Animals were then advanced to tone trials on the next training day. The tone was the same used in the appetitive condition. On tone-signaled trials (again using a 2000 Hz, 90 dB SPL tone), impending foot shocks could be avoided by barpresses during the first 3 sec of tone presentation or escaped by a barpress in the latter 3 sec after the shock was initiated. The shock was delivered as a series of four 250 ms pulses separated by 500 ms periods of no shock. To prevent the animals from adopting a strategy of holding the bar down for excessive amounts of time (thereby avoiding the shock), continuous shock pulses were delivered if the animal failed to release the bar after 5 sec. The trials were separated by 8–12 sec ITIs and a variable 2–6 sec pretone period, during which a barpress resets the pretone period and delayed the initiation of the next trial. One session of avoidance learning consisted of 300 trials (or two hours maximum time), and training continued for ten days. Acquisition days are defined as day one up to the day preceding asymptotic performance. Asymptotic performance was defined as the number of days it took for each animal to reach its own median avoidance rate across all ten days. 

### 2.5. Statistical Analysis

A two-by-two model was used to test restraint by sex comparisons for body weight, all appetitive shaping data, and escape shaping data. To further characterize learning differences in appetitive responding, efficiency ratios (number of correct responses/total barpresses) were assessed. Nonsignificant escape shaping data were collapsed across treatments, and *t*-tests were used to compare sex differences. A repeated measure analysis of variance (RM-ANOVA) tests was used to evaluate body weight data across 3 day bins. Appetitive and avoidance learning were assessed using a repeated measures model with repeated contrasts to look at learning improvement via day-by-day comparisons. Finally, asymptotic performance during avoidance learning was averaged for each group. 

## 3. Results

### 3.1. Body Weight

**Figure 1:**
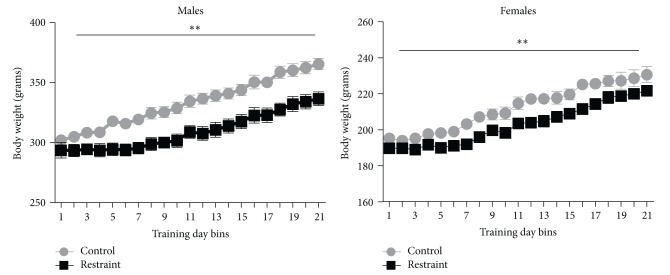
The left side compares the body weights of males across 21 days of restraint with the weights of litter-matched controls. Restraint attenuated weight gain by approximately 7% across the three weeks. The right side compares the body weights of females across 21 days of restraint with the weights of litter-matched controls. Restraint attenuated weight gain by approximately 5% over the three weeks.

Body weight data are presented in Figure 1. Animals were weighed daily for 21 days. Data are presented graphically in three-day bins. There was an overall significant difference between groups for body weight (df (3, 18), *F* = 286.3, *P* < .05). The restrained animals show lower average body weights than their litter-matched controls across all bins with differences averaging approximately 7% for the males (Figure 1) and approximately 5% for the females (Figure 1).

### 3.2. Appetitive Learning Shaping

**Figure 2:**
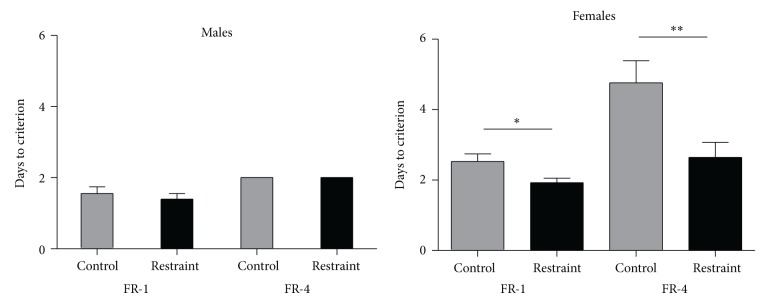
The left side compares the number of days to reach criterion in FR-1 and FR-4 appetitive shaping for control versus restrained males. There were no differences between restrained and control males. The right side compares the number of days to reach criterion in FR-1 and FR-4 appetitive shaping for control versus restrained females. The data show that restrained females reached criterion significantly faster than did control animals and that males reached all criterions faster than females.

Prior to beginning tone-signaled appetitive training, animals were shaped to lever press, initially to criterion on an FR-1 schedule and then to criterion on an FR-4 schedule.  Figure 2 presents the days to criterion acquisition on the two reinforcement schedules for the four groups of subjects. Two 2 × 2 ANOVAs were ran looking at the effect of restraint between sex for the FR-1 task and then the FR-4 task. For the FR-1 task, there was a trend for the effect of restraint (df (1, 49), *F* = 3.897, *P* = .054) and a main effect of sex (df (1, 49), *F* = 15.373, *P* < .05). Restraint facilitated females reaching the FR-1 criterion, while it had minimal effect on males. Males reached the criterion faster than females. For the FR-4 task, there was a significant interaction between restraint and sex (df (1, 46), *F* = 4.264, *P* < .05). Restraint facilitated females in reaching the FR-4 criterion, while it had no effect on males. Males reached the criterion faster than females.

### 3.3. Appetitive Learning-Tone-Signaled Barpressing

**Figure 3:**
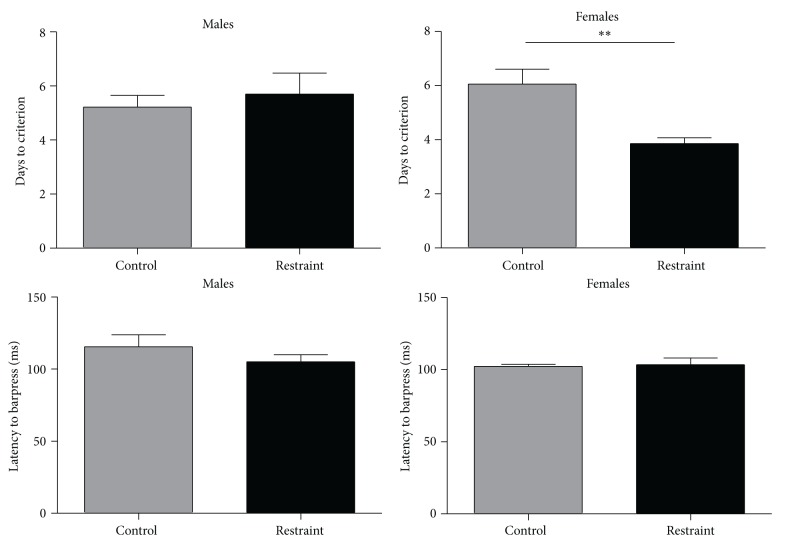
The upper left graph compares the number of days to reach criterion in appetitive training for control versus restrained males. There were no differences between groups. The upper right graph compares the number of days to reach criterion in appetitive training for control versus restrained females. Restrained animals reached appetitive criterion faster than control animals. The lower left graph compares appetitive response latencies for control and restrained males. There were no differences between groups. The lower right graph compares appetitive response latencies for control and restrained females. There were no differences between groups.

After animals achieved criterion on the FR-4 schedule of reinforcement, they were transferred to the tone-signaled task in which they were trained to a criterion of at least 90% CRs over two consecutive days. All subjects achieved the appetitive criterion. For males, the 3-week restrained males did not differ from control males in the rate of acquisition. The 3-week restrained males required, on average, 5.7 ± 0.78 days to achieve criterion. As depicted in the upper left graph of Figure 3, this did not differ from the average of 5.22 ± 0.43 days for the controls (df (1, 17), *F* = .272, *P* > .05). In contrast to the males, there was a difference in the rate at which the restrained and control females achieved criterion. The 3-week restrained females required, on average, 3.86 ± 0.21 days to achieve criterion. As shown in the top right graph of Figure 3, this was significantly faster than the average of 6.06 ± 0.55 days for the controls (df (1, 29), *F* = 12.174, *P* < .05). Between sex comparisons show that male (5.22 ± 0.43 days) and female (6.06 ± 0.55 days) control animals had equivalent rates of acquisition (df (1, 25), *F* = 1.046, *P* > .05), whereas 3-week restrained females achieved appetitive criterion (3.86 ± 0.21 days) significantly faster than their male (5.7 ± 0.78 days) counterparts (df (1, 22), *F* = 7.051, *P* < .05).

Because previous studies demonstrated that females were more efficient at learning in appetitive tasks [10], we also compared the efficiency ratios (correct responses/total barpresses) of males and females over the first two days of tone training. There were no differences in this measure between restrained and control males (df (1, 18), *F* = .204, *P* > .05) or between restrained and control females (df (1, 30), *F* = 3.13, *P* > .05). Because there was no effect of restraint, restrained and control animals were analyzed together to examine sex differences. The average ER across acquisition days 1 and 2 for males was 0.11 ± 0.01, and for females it was 0.17 ± .01, which was significantly different (df (1,18), *F* = 7.16, *P* = .01) with males being less efficient during the acquisition phase of learning. 

For both sexes, average response latencies on the criterion days were comparable. For the males, the lower left graph in Figure 3 shows response latencies averaged 115.5 ± 8.3 and 105.1 ± 4.85 msec for the 3-week and control animals, respectively (df (1, 17), *F* = .490, *P* > .05). Similarly for the females (lower right graph), response latencies averaged 103.4 ± 4.84 and 102.3 ± 1.46 msec for the 3-week and control animals, respectively (df (1, 29), *F* = .026, *P* > .05). 

### 3.4. Aversive Learning-Escape Acquisition

One index of aversive learning is simply whether a subject can be shaped to perform an escape response prior to tone-signaled avoidance training. For this index, we found no significant differences. For males, 100% of the AP-AV males and 89% of the AV males could be shaped. Restrained males, however, showed a slightly different pattern in that only 75% of the restrained males could be shaped to perform the escape response in the AP-AV condition, while 100% could be shaped in the AV condition. For females, 100% of the restrained and control subjects could be shaped to perform an escape response prior to avoidance training in the transfer task (i.e., AP-AV). For AV female subjects, 100% of the restrained and 90% of the control females could be shaped. 

**Figure 4:**
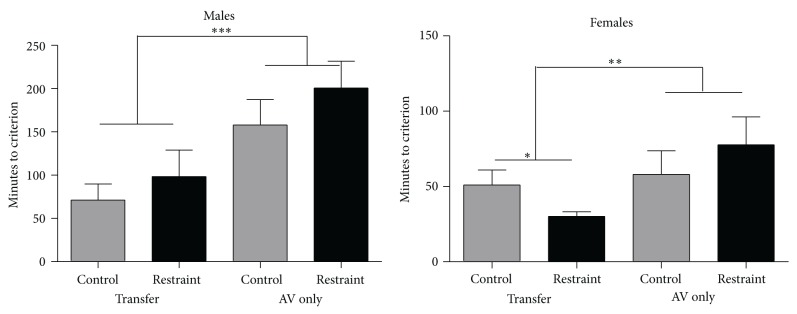
It compares escape shaping performance for transfer and AV-only male and female restrained and control rats. The data show two main effects of sex and task and one interaction of sex by treatment by task: (1) males reached criterion slower than females, (2) transfer animals reached criterion faster than AV-only animals, and (3) female restrained animals reached criterion of the fastest only when they had prior appetitive training. All other restrained animals were slower than their control counterparts.

Another index of the escape shaping response is how long it takes a subject to achieve the criterion.  Figure 4 presents the average number of minutes required to achieve escape criterion for those that did advance to tone-signaled avoidance training. The most apparent difference is that between learning conditions. The graph on the left of Figure 4 shows that AP-AV males (control and restraint) required 84.8 ± 16.76 min to achieve escape criterion, whereas the AV males (control and restraint) averaged 179.75 ± 21.30 min (df (1, 31), *F* = 12.376, *P* < .01). However, there were no differences between restrained and control males. The graph on the right of Figure 4 is the escape learning data for the females. All female animals (control and restrained) that had prior appetitive training (AP-AV) learned the escape response in 40.6 ± 4.69 min. This was significantly faster than the average of 74.6 ± 13.58 min for the AV females (df (1, 58), *F* = 4.79, *P* < .05). Restraint moderately reduced the amount of time to reach criterion in females (*P* = .07) for transfer females. Control females learned the escape response in an average of 54.6 ± 9.3 min, while restrained females averaged 51.1 ± 9.2 min (*P* > .05). 

Between sex analyses revealed that males and females differed substantially in the rate at which they learned the escape response (Figure 4). The female subjects achieved escape criterion in an average of 57.6 ± 6.81 min, while male subjects required 132.12 ± 13.5 min (*t* = 36.25, *P* < .001). Given observations in the literature (for review, see [18]) of sex differences in aversive learning (eyeblink conditioning), we have also compared control male and female subjects in the AV condition to determine the contributions, if any, of restraint and/or prior appetitive training. The control females achieved escape criterion in an average of 61.8 ± 11.37 min, while the control males averaged 114.61 ± 19.86 min, a difference that is statistically significant (*t* = 6.13, *P* < .05).

### 3.5. Aversive Learning-Avoidance Acquisition after Appetitive Training

**Figure 5:**
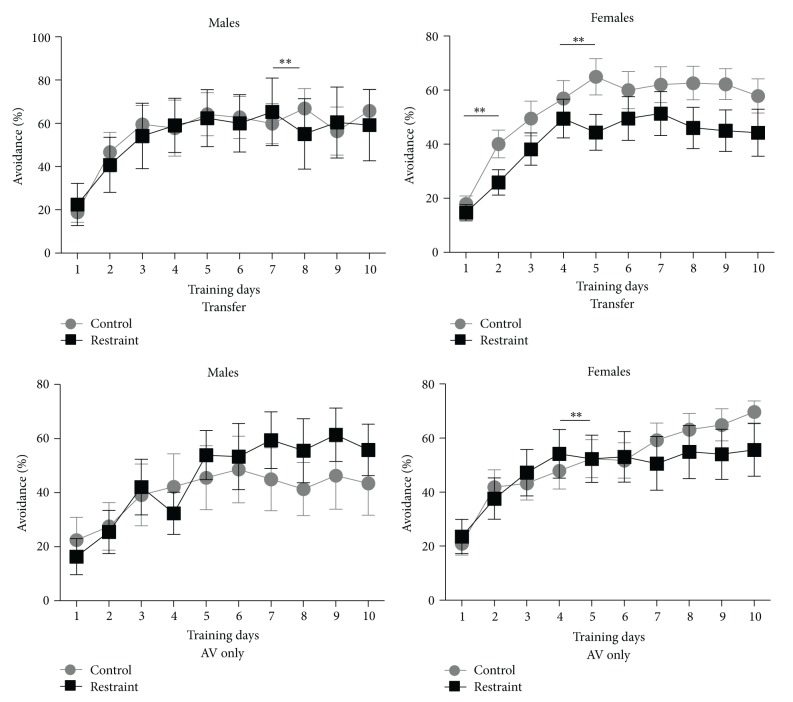
The upper left graph compares avoidance % for restrained transfer males versus control males. Restrained males were not impaired relative to control animals during acquisition but had less stable response patterns after asymptotic training days. The upper right graph compares avoidance % for restrained transfer females versus control females. Restrained females were impaired relative to control animals during acquisition and at the asymptotic transition point. The lower left graph compares avoidance % for restrained aversive-only males versus control males. Restrained males were delayed in reaching asymptotic levels relative to control animals and also had less stable responding patterns. The lower right graph compares avoidance % for restrained aversive-only females versus control females. Restrained females were facilitated in reaching the asymptotic transition point relative to control animals.

**Figure 6:**
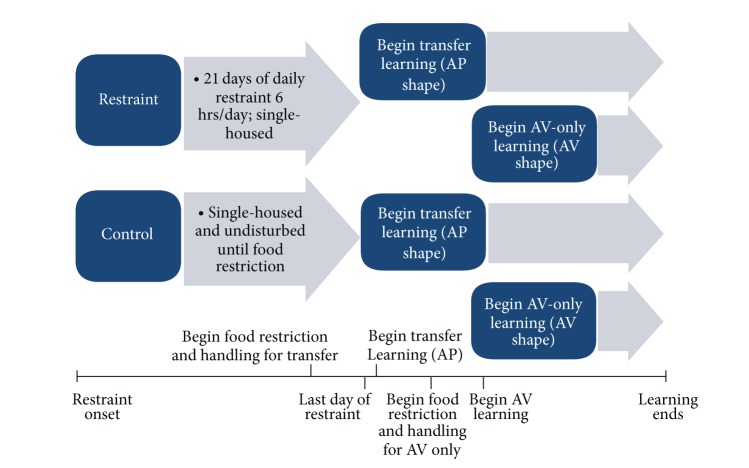
Shows the timeline for the restrained and control animals' progressions into each learning paradigm for male and female animals.

When animals achieved shaping criterion, they were transferred to the aversive phase of the paradigm and trained on a tone-signaled avoidance task. The graph on the upper left in Figure 5 presents avoidance rates for the 3-week restrained and control males. There were no overall differences between groups (df (1, 13), *F* = .020, *P* > .05). The average avoidance rate of the 3-week restrained males (53.90 ± 14.16%) was comparable to that of the controls (55.89 ± 9.44%). Also, there were no descriptive differences in that control and restrained animals reached their median avoidance rate in an average of three days; however, the day-by-day contrast model found a significant difference after asymptotic transition between days seven and eight (df (1, 13), *F* = 10.421, *P* < 0.01).

The graph on the upper right of Figure 5 presents avoidance rates for the 3-week restrained and control females. The overall average avoidance rate for the 3-week restrained females was 40.8 ± 6.8% and was 23% lower than the control average of 53.4 ± 6.0%; however, this difference was not statistically reliable across training (df (1, 28), *F* = 2.52, *P* > .05). Upon closer examination of the data, differences were found between individual training days between groups The control animals acquired the avoidance response at a significantly faster rate from training day one to day two (df (1, 27), *F* = 4.462, *P* < .05) relative to restrained animals, but no other differences were found between acquisition days. Following acquisition, there was a significant difference between groups in avoidance % from training day four to day five, which matched the transition from acquisition to asymptotic performance (df (1, 27), *F* = 7.71, *P* = .01). There were no descriptive differences in asymptotic performance in that control and restrained animals reached asymptote by day four.

A between sex comparison found that overall avoidance learning was comparable for male (55.89 ± 9.44%) and female (53.4 ± 6.0%) controls (df (1, 23), *F* = .069, *P* > .05). The overall average avoidance rate of the 3-week restrained females (40.8 ± 6.8%) was 26% lower than that of the 3-week restrained males (53.90 ± 14.16), but this difference was not statistically reliable (df (1, 17), *F* = 1.220, *P* > .05). However, there were differences in the amount of time to reach the median avoidance performance, as the restrained males reached asymptotic levels one day earlier (day three) than restrained females (day four). While there were no day-by-day contrast differences during asymptotic transition, there were significant differences between females and males after transition at days seven and eight (df (1, 40), *F* = 5.329, *P* < 0.05) and days eight and nine (df (1, 40), *F* = 7.849, *P* < 0.05). 

### 3.6. Aversive Learning-Avoidance Acquisition

Additional animals underwent aversive conditioning without prior appetitive training. The pattern of results for the males was the same as that found for males in the transfer task. The bottom left corner of Figure 5 presents avoidance percentages for 3-week restrained and control males. The overall average avoidance rates for the 3-week restrained animals (45.57 ± 9.57%) and the controls (40.13 ± 11.07%) were comparable (df (1, 15), *F* = .183, *P* > .05). There was a descriptive difference in that aversive-only control males required three days to reach asymptotic performance, while restrained males needed five days; however, there were no significant differences for any day- by-day contrasts. 

The pattern of results for the females was quite different from that found in the transfer task. The lower right graph on Figure 5 presents avoidance percentages for 3-week restrained and control females. There were no significant differences between groups across training (df (1, 27), *F* = .117; *P* > .05). The overall average avoidance rate for the 3-week restrained animals (48.3 ± 8.8%) was quite comparable to that of the controls (51.5 ± 6.02%). Identical to transfer animals, the control and restrained aversive-only females required an average of four days to reach the median avoidance performance. Similar to transfer animals, there is a significant difference on day four versus five, but in the opposite direction. During aversive-only learning, restrained animals had an enhanced rate of responding relative to control animals (df (1, 35), *F* = 4.440, *P* < .05).

In directly comparing male and female subjects, no overall significant differences were detected across training. For both the control (df (1, 25), *F* = 1.269, *P* > .05) and restrained animals (df (1, 17), *F* = .053, *P* > .05), males and females performed comparably. However, there were descriptive differences in the time needed to reach asymptotic performance. Interestingly, there was a group by sex interaction, in that restrained males were the slowest (day five), while control males were the fastest (day three) with all females falling in the middle (day four). The day-by-day contrast model found significant sex differences between days four and five (df (1, 42), *F* = 7.506, *P* < 0.01) and days six and seven (df (1, 42), *F* = 7.049, *P* < .05). All females are comparable on both day-pairs as control animals show slight improvement, while restrained animals show slight decline. In contrast, all males improved with the exception of the control males from day six to seven who show the greatest magnitude of change (declining).

## 4. Discussion

The present experiments have revealed a number of effects of stress and sex differences in indices of both appetitive and aversive instrumental learning. Even though the effects of three weeks of chronic restraint did not result in gross overall deficits in aversive learning, there were several robust and consistent findings that emerged, such as: faster appetitive acquisition for restrained females, reduced avoidance rates in transfer females (acquisition and asymptotic transition), response maintenance fluctuations after asymptotic transition in males, delayed asymptotic responding in restrained males in aversive-only learning, and sex differences in each of the tasks. 

These results support the idea that males and females differ fundamentally in their response to stressors in terms of the stimulus valence (i.e., reward versus footshock), the type of task (i.e., instrumental shaping, AP or AV tone-signaled, or tone-signaled transfer), and the point of learning (i.e., acquisition, transition, or maintenance). Restrained females appear to have an overall enhanced arousal level, which may aid their performance in the appetitive task but interferes with subsequent aversive learning, an effect that is not present in aversive-only females which showed slight facilitation during transition to asymptote. In contrast, males generally appear to have developed a less efficient, but highly responsive learning strategy that appears vulnerable to response maintenance fatigue. This strategy is exacerbated during escape shaping and aversive-only learning when the chronically restrained males are delayed in meeting criterion or asymptotic levels relative to control males and all females. 

### 4.1. Associative Learning: Appetitive Learning

Males achieved criterion levels faster than females on both the FR-1 and FR-4 schedules of reinforcement. This finding is consistent with sex differences in appetitive learning reviewed by [9, 19]. While the paradigms employed in those experiments differed somewhat from those used in the present studies, the results are comparable in that males show an appetitive advantage. This difference was attributed to higher rates of barpressing in males than in females [19]. A second experiment further added that the difference in barpress rate was due to the fact that females were more likely to be engaged in collateral activities, presumably due to a higher overall activity level [10]. In the present experiments, males required fewer days to achieve criterion performance in the FR-1 and FR-4 tasks than females because they were much less likely to exceed the 30- (FR-1) and 40-minute (FR-4) limits established in our protocol for shaping on these reinforcement schedules, which suggest a faster rate of barpressing. 

With respect to acquisition of the tone-signaled appetitive response criterion, control males and females in the present experiments performed comparably. The performance of restrained males was no different from that of the controls. In contrast, restrained females achieved appetitive criterion much faster than restrained males and much faster than controls. A previous study pretrained male rats on an appetitive instrumental task and subsequently chronically restrained them for 21 days and measured its effects on appetitive instrumental learning [12]. They reported that the male rats had deficits in responding for food reward and were unmotivated. In the present experiments, with male rats restrained prior to training, we see no difference in lever pressing, further supporting the notion that male rats are impaired by chronic restraint stress only when it is experienced simultaneously with [11] or after training [11, 12, 20]. Similar to previous reports, the efficiency ratio measures over the first two days of tone training revealed a significant difference, with males being less efficient than females. However, there were no differences in this measure between restrained and control males or between restrained and control females.

The faster rate of appetitive learning in the restrained females relative to controls in the present study is comparable to the effects reported by [21, 22] for learning in a radial maze. Three weeks of stress in females was shown to result in faster acquisition of the radial maze relative to female controls [21], while three weeks of stress in males impaired acquisition relative to male controls [22]. We see no impairment in restrained males in appetitive learning and suggest that this is due to tasks demands and/or the apparent sex differences in the effects of stress on neural systems [23]. Previous studies have shown that chronically stressed male tree shrews were not impaired on hippocampal-dependent or independent learning tasks [24, 25].

### 4.2. Associative Learning: Escape Learning

For both males and females, escape learning was faster in animals with prior appetitive training relative to sex-matched controls without that experience. Most likely, this difference arises from the fact that the animals have learned during the appetitive training that the lever is the only effective response manipulation in the operant chamber. Thus, when the animals are transferred to the aversive learning context, lever pressing has become a part of the animals' behavioral repertoires in that context-elevating the likelihood of a barpress relative to that in animals without the prior appetitive training. In the naïve animals, particularly males, freezing (a species-specific defense reaction; see [26]) would be expected to dominate the animals' (males) activity early in training, interfering with acquisition of the arbitrary instrumental response. 

Sex differences in escape learning were also apparent. Females acquired the escape response substantially faster than males. Importantly, this sex difference was maintained when comparing control animals placed directly into the aversive learning context with no prior appetitive experience, so this difference cannot be solely attributed to either the prior appetitive training or to restraint because the sex difference is seen in AV-only control animals. A good deal of research has focused on sex differences in avoidance behavior (e.g., [27–29]), but relatively little attention has been directed toward investigating sex differences in escape performance. While differences in methodologies employed make direct comparisons somewhat tenuous, the present results are consistent with the shorter barpress escape latencies of female Wistar albino and Long-Evans hooded rats relative to strain-matched males reported by [30]. Several factors, such as: developmental gonadal influences, appropriate weight gain, differences in sensitivity and/or reactivity to the shock, higher baseline levels of activity in females (see [18] or [9] for review), and sexually dimorphic amygdalar responsiveness [31] have been suggested to potentially account for such sex differences in aversive learning. Presumably, one or all of these factors could account for the sex differences in escape learning reported here. 

### 4.3. Discrimination Avoidance Learning-Transfer Task

When confronted with a transfer from appetitive to aversive conditioning, the 3-week restrained females were impaired in aversive learning even though they had shown facilitation during escape response learning, suggesting no lack of motivation to avoid. Nevertheless, restrained females had reduced rates of avoidance acquisition. Because the restrained animals performed comparably on the acquisition of the avoidance task when it did not follow the appetitive training, and control animals were equivalent regardless, this suggests that chronic restraint stress may have been more strongly impacting systems involved in reassigning motivational value to the lever press. The transfer task is a more cognitively taxing task because it involves the retrieval and subsequent transfer of information to cortical processes in order to reevaluate the learning strategy and engage decision-making skills. This reevaluation process may result in proactive interference as the behavior is not elicited reflexively but is contingent upon performance and its outcome [32]. Specifically, the cognitive task of reassigning the stimulus valence to the instrumental performance may have been impacted by chronic stress. The basolateral amygdala (BLA), which receives motoric information from the ventral striatum and has been hypothesized to make cue-specific assignments to conditioned stimuli [33, 34], may have been experiencing competitive interference and unable to relay information to the cortex as accurately as control animals. 

One caveat is that these other studies used only males, and since we did not see the same effect in our males, this suggests that either (1) the magnitude and/or rate of the effect is larger in females or (2) the order in which neural systems are impacted varies between sex. Our body weight indices of the stressor manipulation suggest that the magnitude of the effect is equivalent or greater in the males, so it seems unlikely that the effect is larger or more meaningful to females. Alternatively, the notion of variance between sexes in the order of neural systems impacted by chronic stress has support in the studies looking at the interaction of chronic stress and gonadal hormone signaling (see [9, 35]). Studies have suggested both gonadal organizational differences and/or systemic signaling differences accounting for the order in which neural systems are impacted. 

In similar fashion to females, there were no overall differences between groups for male rats in the transfer task. In contrast, males did not show the same pattern of results on the day-by-day contrasts as there were no differences between groups during avoidance acquisition or the day of transition to asymptotic response levels. However, there were reduced response rates from day seven to day eight. Given that this effect is postasymptotic transition, it is likely impacting response maintenance issues, resulting in transient levels of fatigue. Fatigue has been linked to hippocampal impairment [36, 37] and suppressed levels of hippocampal brain-derived neurotrophic factor (BDNF) mRNA [38], an area that has been shown to be important in multiple learning and memory tasks and motivation processing. Importantly, three-week restraint stress has been shown to negatively impact the hippocampus in males [5, 39], but the extent to which the hippocampus may be involved in instrumental transfer learning is not well characterized. 

Transfer males and females were comparable in their overall avoidance % across training, but control males were faster at reaching their asymptotic levels (day 3) than females were (day 4). There were also differences in the day-by-day contrast after the animals reached asymptotic levels (i.e., day seven versus eight and day eight versus nine). Although the restrained females were trending at a lower response rate, all females were stable across day seven to eight; however, the males showed a different pattern. While all males responded comparably on day seven, the restrained animals had a marked decrease on day eight. Interestingly, we see a similar pattern that emerges when looking at the next day (nine), except this time it was the control males that reduced their responding, while restrained males and all females maintained stable response patterns. Taken together, this data suggests that males are highly motivated to respond but have greater response fluctuations from day to day, which may be an indicator of transient response fatigue. In contrast, females are more likely to experience effects on acquisition or at the transition to asymptote, but after transition they maintain a more stable response pattern. In fact, the off-bar shock index (data not shown) further supports this conclusion. When looking at the sum total of the off-bar shocks received, males in all four groups had 1,378 relative to the females' 567, a difference that is significant (*t* = 61.44, *P* < .001). Given that there were no overall differences in aversive learning, these data indicate a more subtle dichotomy between sexes and the impact of chronic stress on acquiring an instrumental response versus efficiently maintaining the response. 

### 4.4. Associative Avoidance Learning-Aversive-Only Task

The aversive-only restrained females performed overall comparably to control animals across days. In contrast to transfer females, there was no effect on acquisition; however, the day-by-day contrast model revealed a significant difference of the day immediately preceding asymptote. Interestingly, on day four, the restrained animals had an enhanced rate of responding relative to control animals, but on day five the two groups were equivalent. The overall pattern of effects due to chronic restraint in females indicates an enhanced responsiveness in the simple associative tasks, which suggests heightened arousal levels. Given that there were no subsequent contrast differences, their response pattern appears to have stabilized at the transition point.

Consistent with females, there was no overall significant difference between groups. In contrast to females, there were no day-by-day contrast effects; however, chronic stress had a larger impact on the males' performances in reaching asymptotic levels. Restrained males had delayed asymptotic response levels. Given that there were no differences in overall or day-by-day contrast responding, it is likely that the stressor load exacerbated the high level of responsiveness of males in the aversive-only task, resulting in delayed asymptotic levels. In fact, the restrained AV-only males did show significantly more off-bar shocks relative to control AV-only males (df (1, 19), *F* = 4.950, *P* < .05). 

Consistent with previous data, there were no overall differences between males and females. However, there was an interaction of sex by group in days to reach asymptotic levels. Control males reached asymptotic levels the fastest (day three), while all females were in the middle (day four), and restrained males were the slowest (day five). There were also significant contrast differences between days four and five, in addition to day six versus seven which revealed an interaction effect that parallels the asymptotic data. Control males and females had slight improvements in responding from day four to day five, while the restrained animals move in opposite directions. Restrained females are significantly higher than restrained males on day four, but their response rates are equivalent by day five (the males asymptotic transition) because the males increased their responding. At a later training day (six versus seven), we see that the restrained males have increased further from their prior enhancements, but that control males significantly decreased their responding. This pattern is consistent with the sex differences in the transfer task, where male responding patterns are less stable postasymptotic transition than female patterns, which remain consistent. 

## 5. Conclusion

The current experiments have demonstrated a remarkable sex difference in response to chronic stress that when compared to the results of the studies examining spatial learning or fear conditioning would suggest that task selection is important for revealing possible neural correlates. In fact, studies have shown neuromolecular correlates of sex by task selection, such as region-specific elevations in Fos levels [40]. Consequently, different learning tasks may be more sensitive in testing one sex versus the other. 

There are also a few limitations of this study to take into account. Because of the limited set of studies utilizing the instrumental transfer paradigm, the generalizability to other studies is diminished. However, we feel this is also a strength of this study as it provides insights into higher level cognitive constructs and attempts to advance the field of stress and sex effects on learning. We contend that more research utilizing similar paradigms is warranted. Also, the chronicity of the restraint paradigm may be impacting neural structures mediating effects that are “downstream” of the primary stressor, such as anhedonia or sensitivity to satiety or pain signals. The current study did not assess the impact on sensory or physiological systems. Finally, because we elected to control for appetitive learning time to compare the transfer and aversive-only learning, this may have reduced the magnitude of the effect that restraint had on aversive-only learning in all animals. 

The results of this study and other studies lend support to a theoretical view of chronic stress that takes into account context-deviation specificity [41]. Context-deviations encompass a wide range of parameters, including stressor type, duration frequency, and the location and surrounding environment the stressor is encountered. Additionally, previous exposure to other stressors and/or repeated exposure to an ongoing stressor can also increase the magnitude of the effect. Given the reported differences seen on the effect of the timing of stressor onset (before, during, or after conditioning), it is clearly not just the complexity of the stimulus that determines the response, as early responses impact subsequent responses. More research is needed to resolve the psychosocial and neural implications of these sex differences, but we suggest that the learning paradigms employed in the present experiments will prove useful in revealing important nuances in these differences.
